# Growth curves for Turkish Girls with Turner Syndrome: Results of the Turkish Turner Syndrome Study Group

**DOI:** 10.4274/jcrpe.2023

**Published:** 2015-08-31

**Authors:** Feyza Darendeliler, Ediz Yeşilkaya, Abdullah Bereket, Firdevs Baş, Rüveyde Bundak, Erkan Sarı, Banu Küçükemre Aydın, Şükran Darcan, Bumin Dündar, Muammer Büyükinan, Cengiz Kara, Mümtaz M. Mazıcıoğlu, Erdal Adal, Ayşehan Akıncı, Mehmet Emre Atabek, Fatma Demirel, Nurullah Çelik, Behzat Özkan, Bayram Özhan, Zerrin Orbak, Betül Ersoy, Murat Doğan, Ali Ataş, Serap Turan, Damla Gökşen, Ömer Tarım, Bilgin Yüksel, Oya Ercan, Şükrü Hatun, Enver Şimşek, Ayşenur Ökten, Ayhan Abacı, Hakan Döneray, Mehmet Nuri Özbek, Mehmet Keskin, Hasan Önal, Nesibe Akyürek, Kezban Bulan, Derya Tepe, Hamdi Cihan Emeksiz, Korcan Demir, Deniz Kızılay, Ali Kemal Topaloğlu, Erdal Eren, Samim Özen, Hüseyin Demirbilek, Saygın Abalı, Leyla Akın, Beray Selver Eklioğlu, Sultan Kaba, Ahmet Anık, Serpil Baş, Tolga Ünüvar, Halil Sağlam, Semih Bolu, Tolga Özgen, Durmuş Doğan, Esra Deniz Çakır, Yaşar Şen, Nesibe Andıran, Filiz Çizmecioğlu, Olcay Evliyaoğlu, Gülay Karagüzel, Özgür Pirgon, Gönül Çatlı, Hatice Dilek Can, Fatih Gürbüz, Çiğdem Binay, Veysel Nijat Baş, Celal Sağlam, Davut Gül, Adem Polat, Cengizhan Açıkel, Peyami Cinaz

**Affiliations:** 1 İstanbul University Istanbul Faculty of Medicine, Department of Pediatric Endocrinology, İstanbul, Turkey; 2 Gülhane Military Medicine Academy, Department of Pediatric Endocrinology, Ankara, Turkey; 3 Marmara University Faculty of Medicine, Department of Pediatric Endocrinology, İstanbul, Turkey; 4 Ege University Faculty of Medicine, Department of Pediatric Endocrinology, İzmir, Turkey; 5 Katip Çelebi University Faculty of Medicine, Department of Pediatric Endocrinology, İzmir, Turkey; 6 Konya Training and Research Hospital, Clinic of Pediatric Endocrinology, Konya, Turkey; 7 On Dokuz Mayıs University Faculty of Medicine, Department of Pediatric Endocrinology, Samsun, Turkey; 8 Erciyes University Faculty of Medicine, Department of Pediatric Endocrinology, Kayseri, Turkey; 9 Kanuni Sultan Süleyman Training and Research Hospital, Clinic of Pediatric Endocrinology, İstanbul, Turkey; 10 Inönü University Faculty of Medicine, Department of Pediatric Endocrinology, Malatya, Turkey; 11 Necmettin Erbakan University Faculty of Medicine, Department of Pediatric Endocrinology, Konya, Turkey; 12 Yıldırım Beyazıt University Faculty of Medicine, Department of Pediatric Endocrinology, Ankara, Turkey; 13 Gazi University Faculty of Medicine, Department of Pediatric Endocrinology, Ankara, Turkey; 14 Dr. Behçet Uz Children Hospital, Clinic of Pediatric Endocrinology, İzmir, Turkey; 15 Pamukkale University Faculty of Medicine, Department of Pediatric Endocrinology, Denizli, Turkey; 16 Atatürk University Faculty of Medicine, Department of Pediatric Endocrinology, Erzurum, Turkey; 17 Celal Bayar University Faculty of Medicine, Department of Pediatric Endocrinology, Manisa, Turkey; 18 Yüzüncü Yıl University Faculty of Medicine, Department of Pediatric Endocrinology, Van, Turkey; 19 Harran University Faculty of Medicine, Department of Pediatric Endocrinology, Şanlıurfa, Turkey; 20 Uludağ University Faculty of Medicine, Department of Pediatric Endocrinology, Bursa, Turkey; 21 Çukurova University Faculty of Medicine, Department of Pediatric Endocrinology, Adana, Turkey; 22 İstanbul University Cerrahpaşa Faculty of Medicine, Department of Pediatric Endocrinology, İstanbul, Turkey; 23 Kocaeli University Faculty of Medicine, Department of Pediatric Endocrinology, Kocaeli, Turkey; 24 Osmangazi University Faculty of Medicine, Department of Pediatric Endocrinology, Eskişehir, Turkey; 25 Karadeniz Technical University Faculty of Medicine, Department of Pediatric Endocrinology, Trabzon, Turkey; 26 Dokuz Eylül University Faculty of Medicine, Department of Pediatric Endocrinology, İzmir, Turkey; 27 Diyarbakır Training and Research Hospital, Clinic of Pediatric Endocrinology, Diyarbakır, Turkey; 28 Gaziantep University Faculty of Medicine, Department of Pediatric Endocrinology, Gaziantep, Turkey; 29 Düzce University Faculty of Medicine, Department of Pediatric Endocrinology, Düzce, Turkey; 30 Selçuk University Faculty of Medicine, Department of Pediatric Endocrinology, Konya, Turkey; 31 Keçiören Training and Research Hospital, Clinic of Pediatric Endocrinology, Ankara, Turkey; 32 Süleyman Demirel University Faculty of Medicine, Department of Pediatric Endocrinology, Isparta, Turkey; 33 Kayseri Training and Research Hospital, Clinic of Pediatric Endocrinology, Kayseri, Turkey

**Keywords:** Turner syndrome, growth charts, body mass index charts, Turkish children

## Abstract

**Objective:**

Children with Turner syndrome (TS) have a specific growth pattern that is quite different from that of healthy children. Many countries have population-specific growth charts for TS. Considering national and ethnic differences, we undertook this multicenter collaborative study to construct growth charts and reference values for height, weight and body mass index (BMI) from 3 years of age to adulthood for spontaneous growth of Turkish girls with TS.

**Methods:**

Cross-sectional height and weight data of 842 patients with TS, younger than 18 years of age and before starting any therapy, were evaluated.

**Results:**

The data were processed to calculate the 3rd, 10th, 25th, 50th, 75th, 90th and 97th percentile values for defined ages and to construct growth curves for height-for-age, weight-for-age and BMI-for-age of girls with TS. The growth pattern of TS girls in this series resembled the growth pattern of TS girls in other reports, but there were differences in height between our series and the others.

**Conclusion:**

This study provides disease-specific growth charts for Turkish girls with TS. These disease-specific national growth charts will serve to improve the evaluation of growth and its management with growth-promoting therapeutic agents in TS patients.

## INTRODUCTION

Turner syndrome (TS) is a common chromosomal disorder occurring in 1:2500 female live births. The most common presenting symptom in girls with TS is short stature which is mild during early childhood but becomes more notable with age. Untreated females are reported to be approximately 18-20 cm shorter than the general population (1,2,3,4).

Growth curves are widely used in pediatric practice and are very important tools for the evaluation of child health (5,6,7,8,9). The curves proposed by international health organizations, such as the World Health Organization (10) and the Centers for Disease Control and Prevention (11), provide growth evaluation in children belonging to different ethnicities and socio-economic conditions. However, children with specific diseases (including TS) and having a specific growth pattern cannot be well assessed with the curves designed for healthy children. Thus, disease-specific growth charts are required for better evaluation of growth and response to growth-promoting therapies in certain diseases.

There are growth curves for girls with TS from different countries which are affected by ethnicity and genetic factors (12,13,14,15,16,17,18,19,20,21,22,23,24). Preliminary studies have revealed that patients with TS have a high incidence of obesity. Weight-associated height references are therefore required in this area (13,20,24,25). Currently, there are no population-specific growth standards that allow monitoring of the growth of Turkish TS patients of different ages or provide a basis for a fairly accurate prediction of their adult height.

We therefore undertook a collaborative study to construct growth charts and reference equations for age-matched height, weight and body mass index (BMI) values from 3 years of age to adulthood for spontaneous growth of Turkish girls with TS.

## METHODS

This study on the evaluation of nationwide data of patients with TS in Turkey, the details of which have been reported previously, was conducted by the Turkish Turner Syndrome Study Group (26).

### Patients and Data Collection

A total of 842 patients with TS, younger than 18 years of age, who presented to 35 different centers in Turkey between 1984 and 2014, were enrolled in this cross-sectional study. The study was approved by the Ethics Committee of Gülhane Military Medical Academy.

A Case Recording Form (CRF) was created which contained data on height and weightat first admission. Physicians working in outpatient clinics for TS patients were asked to fill in these CRF forms. The data were collected by physicians who were chosen to be responsible for the registration at each center. The data were assessed by four physicians (EY, FD, ES and PC) experienced in TS and were uploaded to the online web registry system located in the web site www.favorsci.org by van expert on electronic CRF preparation (CA). Data entered in the registry was also checked for consistency by a research assistant (EY).

A standard 30-cell karyotype analysis from peripheral blood was made in all patients. The diagnosis of TS was confirmed by reviewing all the reported karyotypes of cultured peripheral blood lymphocytes. Karyotypes of patients were assigned to numerical, structural and both numerical and structural abnormalities by a geneticist (DG).

Height and weight measurements taken according to standard techniques and recorded at presentation were collected retrospectively. After excluding the patients who were younger than 3 years of age and those with a history of growth-promoting treatment (such as growth hormone, estrogen, oxandrolone), we enrolled a total of 717 patients in the study.

The height values were interpolated individually to the nearest of the defined ages by expressing the measured height values as standard deviation score (SDS) using the Turkish references for height. The weight measurements were interpolated to full age values using the same method as height-for-age (27). BMI was calculated by dividing the weight by the square height or length (kg/m2). The obtained reference curves for height-for-age, weight-for-age and BMI-for-age of girls with TS were compared with those of healthy Turkish girls (27). For each defined age, 3rd, 10th, 25th, 50th, 75th, 90th and 97th percentile values were calculated by a statistician (MMM). For the specific percentile line, age ±6 month data were taken.

### Statistical Analysis

Construction of the centile curves was performed with the LMS Chart Maker Pro version 2.3 software program (The Institute of Child Health, London), which fits smooth centile curves to reference data using the LMS method. The smoothed centile curves of BMI were constructed by the LMS method. This method summarizes percentiles at each age based on the power of age-specific Box-Cox power transformations that are used to normalize data. These three quantities depend on age. The final curves of percentiles are produced by three smooth curves representing Lambda; skewness (L), Mu; median (M) and Sigma; coefficient of variation (S). The LMS transformation equation is: X=M (1+LSz)1/L L≠0 or X=M exp (Sz) L=0 where X is the physical measurement and z is the z-score that corresponds to the percentile. The key task of the transformation was to estimate parameters L, M and S. With estimates of L, M and S, values of X are connected to the values of z through the above equation. The percentile is obtained from a normal distribution table where the z-score corresponds to the percentile of interest.

## RESULTS

Karyotype analyses of the patients are shown in Table 1. Half of the patients had a 45,X chromosome constitution. Other karyotypes were as follows: 10.8%, 10.1% and 9.5% for 45,X/46,XX, 46,X,i (Xq) and 45,X/46,X,i (Xq), respectively.

### Height-for-Age

**Figure 1:**
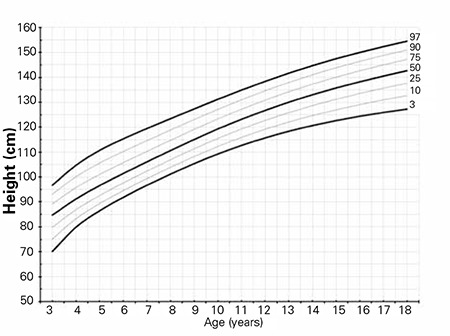
Height percentiles of Turkish Turner syndrome girls

**Figure 2:**
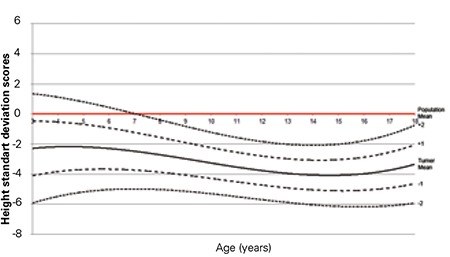
Mean change in height standard deviation scores of Turkish Turner syndrome girls

**Figure 3:**
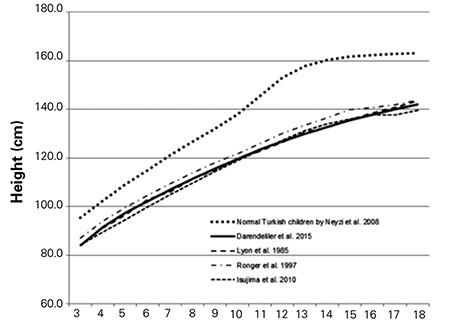
The comparison of the mean height of Turner syndrome girls with that of other nationalities and age-matched healthy Turkish children

The L, M, S values and percentile values, the height growth chart and the mean change in height SDS for Turkish girls with TS are shown in Table 2, Figures 1 and 2, respectively. The comparison of the mean height of TS girls with that of other nationalities and with an age-matched Turkish healthy population is shown in Figure 3.

All mean height values in TS patients were lower in comparison to the mean values of healthy girls. The mean height values from 3 years of age were lower than that of the general population and got lower by age. While height was under -2 SD at age 3 years, this decline got closer to -4 SD at about 13 years of age. From the age of 13, we observed that there was an increase of approximately 2.5-3 cm per year in height without pubertal peak. Around the ages of 14-15, the height distribution showed that -1 SD and -2 SD values for Turner girls nearly equaled the -5 SD and -6 SD values for the age-matched healthy girls, respectively.

The mean height of Turkish girls with TS, computed at 18.0 years of age, was found to be 141.9±6.9 cm in this cohort study, representing a deficit of about 21.2 cm according to the population mean (163.1±5.9 cm).

### Weight-for-Age

**Figure 4:**
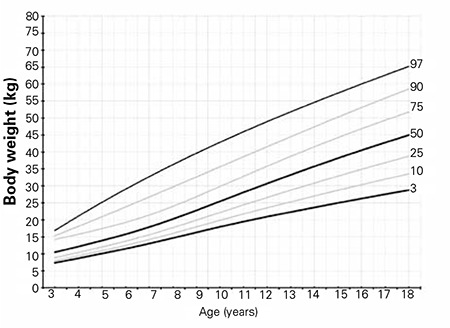
Body weight percentiles of Turkish Turner syndrome girls

**Figure 5:**
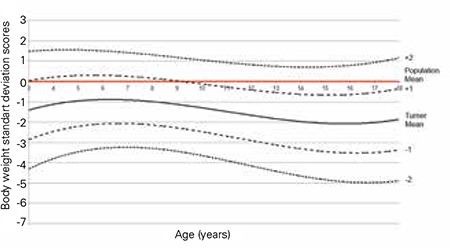
Mean change in weight standard deviation scores of Turkish Turner syndrome girls

**Figure 6:**
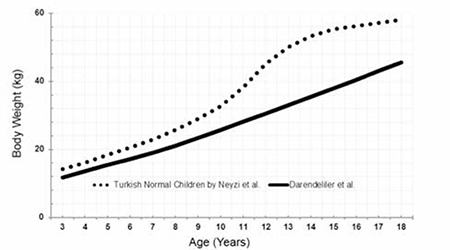
The comparison of the mean weight of Turkish Turner syndrome girls with the age-matched healthy population

The L, M, S values and percentile values, the weight growth chart and the mean change in weight SDS for the Turkish girls with TS are shown in Table 3, Figures 4 and 5, respectively. The mean weight of Turkish girls with TS as compared to the mean weight of healthy Turkish girls is shown in Figure 6. All mean weights at different ages were lower in comparison to healthy girls. Mean weight SDS values from 3 years of age were lower than those of the general population and gradually decreased by age. At ages 16-17 years, the mean weight-for-age of TS girls was lower than the reference -2 SD, whereas thereafter, the weight distribution showed that +2 SD and -2 SD for Turner girls nearly approached the reference +1 SD and -5 SD, respectively (27).

The 50th percentile of weight, computed at 18.0 y of age, was 45.0 kg and below that of the healthy population in this cohort study.

### Body Mass Index-for-Age

**Figure 7:**
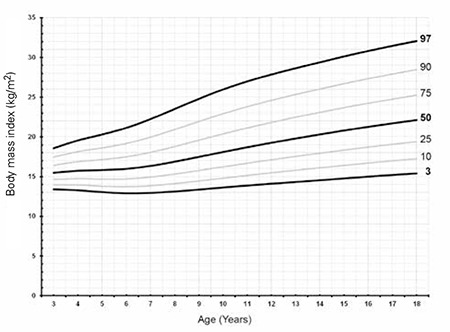
Body mass index percentiles of Turkish Turner syndrome girls

**Figure 8:**
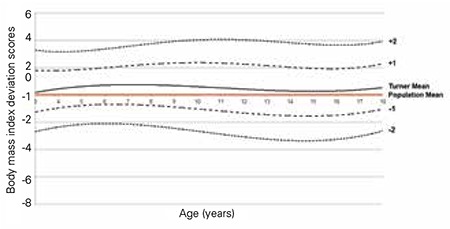
Mean change in body mass index standard deviation scores of Turkish Turner syndrome girls

**Figure 9:**
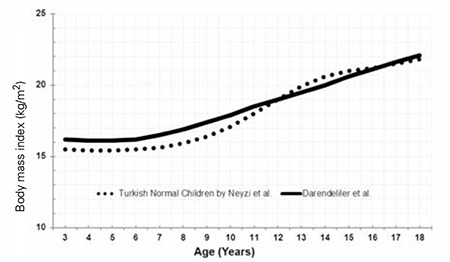
The comparison of the mean body mass index of Turkish Turner syndrome girls with the age-matched healthy population

The BMI distributions of the Turkish girls with TS are shown in Table 4, Figures 7 and 8. The mean BMI of Turkish girls with TS as compared to the mean BMI of healthy Turkish girls is shown in Figure 9.

The mean BMI distribution for TS at 3 years of age nearly approached the mean of the reference population and gradually increased by age. We also observed a plateau after 6 years of age, whereas thereafter, it always remained higher than the reference ranges. The BMI distribution showed that the +1 SD for Turner girls nearly equals the +2 SD of the reference girls.

## DISCUSSION

This study presents the growth data and growth curves of Turkish TS children. Three important considerations led us to realize the need for TS-specific growth charts for the children of our community.

Firstly, growth hormone (GH) and/or oestrogen, oxandrolone therapies, in addition to their favorable metabolic effects, are recommended to accelerate growth and final height in TS patients. However, supra-physiological GH dosages are usually necessary to induce height acceleration in these girls (28). The response to GH is rather variable depending on the different treatment protocols such as dosing regimens, adjuvant therapy with oxandrolone, age at start of GH therapy and management of puberty (29). Besides providing accurate information for the monitoring of growth in TS girls, TS-specific growth charts are also important in order to elucidate the response to growth-promoting therapies.

Secondly, many studies indicate that there may be associated autoimmune disorders such as Hashimoto thyroiditis and celiac disease that may further inhibit the growth of TS patients. Short stature and/or low growth velocity can be the primary or sole manifestation of these disorders. Thus, evaluating the growth pattern may also be helpful in establishing the early diagnosis and understanding the pathogenesis of associated disorders which might influence growth (30,31). The use of population-specific growth charts for the monitoring of growth in TS girls is therefore also recommended to detect the presence of an additional pathology (12).

Thirdly, girls with TS are prone to obesity because of excessive weight gain with a central or android fat distribution, particularly. This predisposition to obesity may be exaggerated in appearance by the characteristics of TS patients such as shield-like chest, stocky build and short stature with relatively short legs. The patients should be evaluated regularly with appropriate counseling to avoid obesity (32).

The growth pattern of patients with TS is characterized by intrauterine growth retardation, slow growth during the first three years of life, impaired pre-pubertal growth and lack of a pubertal growth spurt. In our cohort, the results indicated that height velocity declines after the first year of life progressively and is below -2 SD around 3-4 years of age. There is no significant pubertal growth spurt and the height SDS approaches -4 SD around age 12 years. However, the children continue to grow and reach a height SDS of -2.6 SD at 18 years of age. This further gain in height SD is also observed in patients without any pubertal development due to two possible reasons. Firstly, hormones other than gonadal steroids contribute to the normal pubertal growth spurt in girls. Secondly, there may be some residual function of the ovaries in Turner patients at the time of puberty and bone is more sensitive to estrogens than is breast tissue. The spurt in height growth often occurring as the first sign of puberty in normal girls is a finding which is in support of this latter theory (14,29,33,34).

Ethnicity and genetic factors are important determinants of the growth curves for girls with TS. For instance, studies reveal that Japanese patients were shorter than the European patients (35). Therefore, country-specific growth curves should be generated. The comparison of our growth charts to two previously created percentile curves (by Lyon et al (16) and Rongen-Westerlaken et al (35)) revealed that the mean height percentile of our patients is almost identical to that reported by the first group of researchers but significantly lower than that reported by the second one.

Mean height at age 18 years was 141.9±6.9 cm in the present study. A previous study on 110 untreated Turkish Turner subjects reported a final height of 141.6±7.0 cm at 23 years of age (1). The slightly higher final height in the present study might reflect a secular trend or might be due to the small number of subjects of age 18 in our study.

BMI provides more information than weight-for-age and should be used as an index of adiposity in children. We constructed disease-specific charts for both weight-for-age and BMI in girls with TS. Several studies have shown that in general, the mean and SD values for weight in TS girls tend to increase with age. In our study, we observed that the distribution of BMI values gradually increased with age and that a +1 SD value for Turner girls nearly equals a +2 SD value for the reference for normal Turkish girls. Further studies are needed to reveal which method is more effective in interpreting the relationship between weight and height in girls with TS. In this study, our results indicate that the mean SD value for BMI increased around 2 years of age and remained higher than the general population (35).

The limitation of our study is its cross-sectional design and we have no data regarding growth velocity and spontaneous pubertal status. Moreover, it is not known whether subjects showing no signs of puberty at the time of registration will develop spontaneous puberty later. This is an important topic, because preliminary studies revealed that those with spontaneous puberty, from 12 years of age onward, are significantly taller than those without puberty, although pubertal development and growth spurt do not seem to affect final adult height (7). Nevertheless, a longitudinal study on spontaneous growth in TS would not be possible for ethical reasons.

In conclusion, we have presented cross-sectional data on the spontaneous growth of a large number of Turkish girls with TS. These growth charts will be useful in following the natural growth, weight gain and also the growth-promoting effects of medical agents in these children.

## Figures and Tables

**Table 1:**
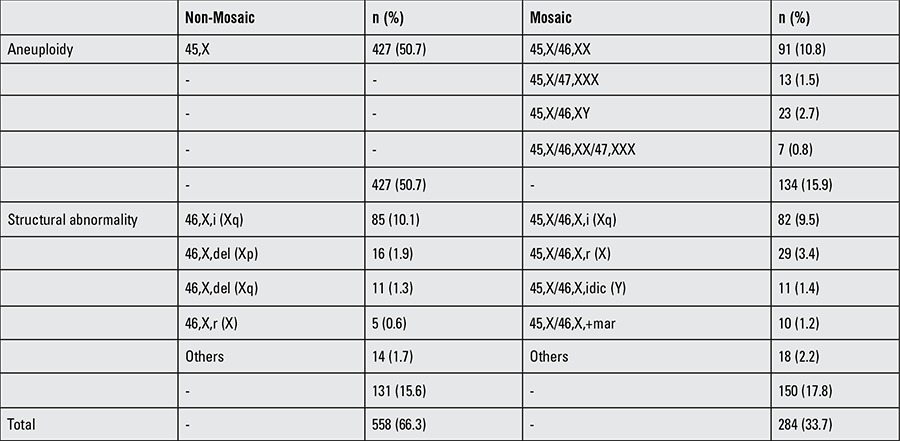
Distribution of Turner syndrome patients according to karyotype (n=842)

**Table 2:**
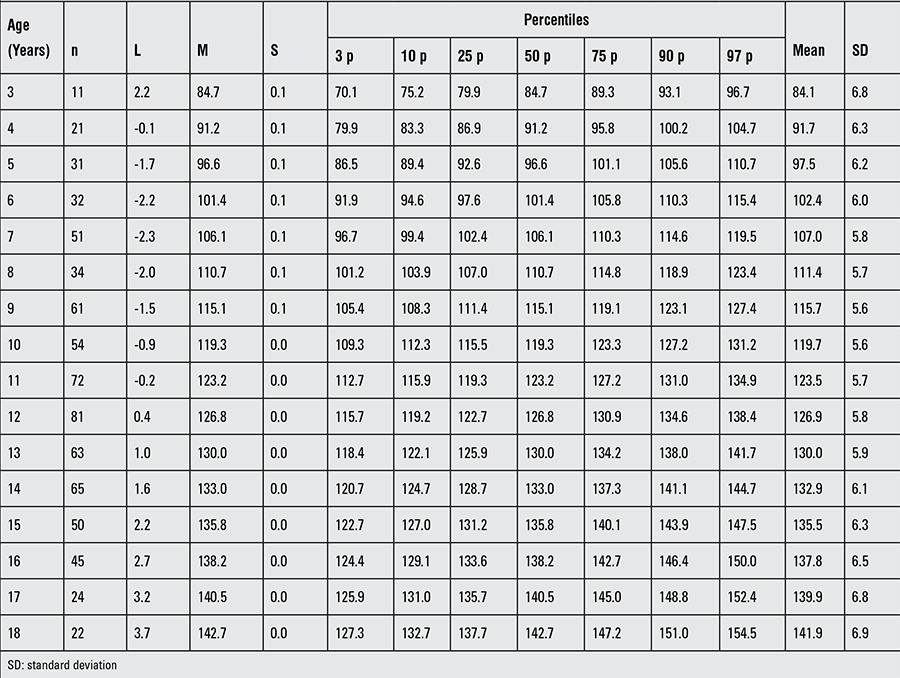
Height of patients with Turner syndrome at different ages

**Table 3:**
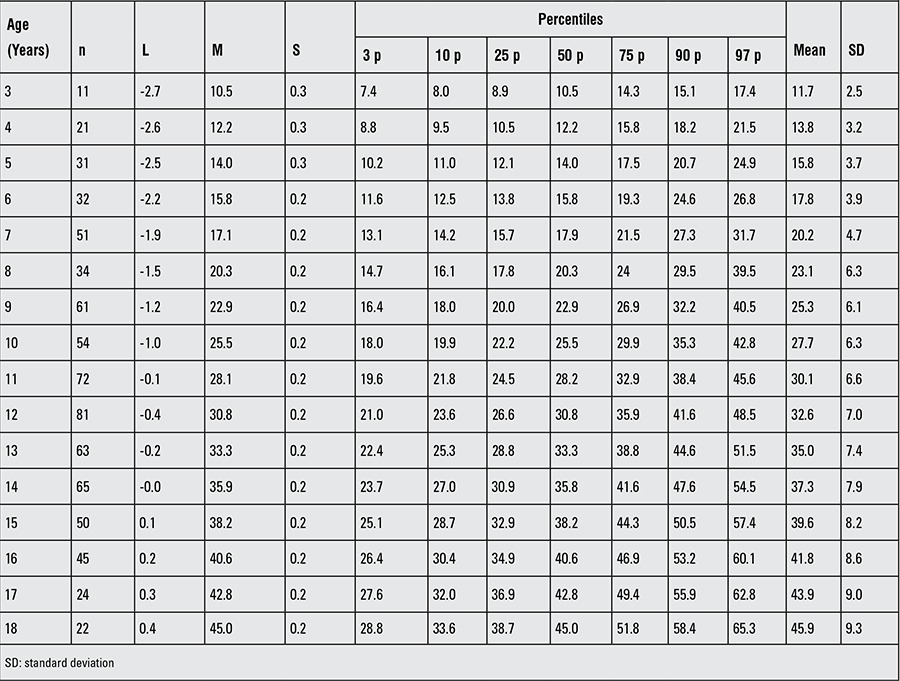
Body weight of patients with Turner syndrome at different ages

**Table 4:**
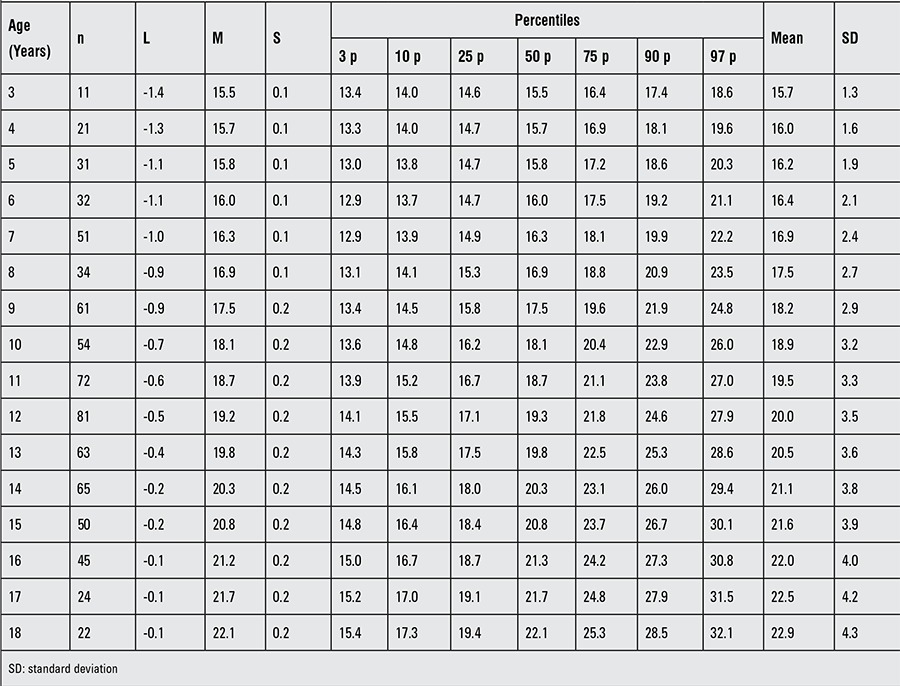
Body mass index of patients with Turner syndrome at different ages
